# Photochemical and Photophysical Properties of Phthalocyanines Modified with Optically Active Alcohols

**DOI:** 10.3390/molecules200813575

**Published:** 2015-07-24

**Authors:** Aline A. Ramos, Francisco B. Nascimento, Thaiza F. M. de Souza, Alvaro T. Omori, Tânia M. Manieri, Giselle Cerchiaro, Anderson O. Ribeiro

**Affiliations:** Centro de Ciências Naturais e Humanas—CCNH, Universidade Federal do ABC—UFABC, Avenida dos Estados, 5005, Bairro Bangu, Santo André—SP 09210-190, Brasil; E-Mails: linearamos@gmail.com (A.A.R.); francisco.comunic@gmail.com (F.B.N.); thaizamenegassi@gmail.com (T.F.M.S.); alvaro.omori@ufabc.edu.br (A.T.O.); tania.manieri@gmail.com (T.M.M.); gicerchiaro@gmail.com (G.C.)

**Keywords:** chiral phthalocyanines, enzymatic resolution, photodynamic therapy, photosensitizer, dyes

## Abstract

Three phthalocyanine derivatives were synthesized and characterized: one modified with a racemic mixture of 1-(4-bromophenyl)ethanol and two other macrocycles modified with each one of the enantioenriched isomers (*R*)-1-(4-bromophenyl)ethanol and (*S*)-1-(4-bromophenyl)ethanol. The compounds were characterized by ^1^H-NMR spectroscopy, mass spectrometry, UV-Vis absorption, and excitation and emission spectra. Additionally, partition coefficient values and the quantum yield of the generation of oxygen reactive species were determined. Interestingly, the phthalocyanine containing a (*R*)-1-(4-bromophenyl)ethoxy moiety showed higher quantum yield of reactive oxygen species generation than other compounds under the same conditions. In addition, the obtained fluorescence microscopy and cell viability results have shown that these phthalocyanines have different interactions with mammary MCF-7 cells. Therefore, our results indicate that the photochemical and biological properties of phthalocyanines with chiral ligands should be evaluated separately for each enantiomeric species.

## 1. Introduction

Phthalocyanines are a highly conjugated macrocycles with good thermal and chemical stability, properties that allow them to be applied in many different fields, such as use as photosensitizers in photodynamic therapy [1].

However, because of the phthalocyanines’ planarity, a high self-aggregation in polar environments is observed [2], which reduces their solubility and significantly affects their photochemical and photophysical properties in these media. To circumvent this problem, the preparation and characterization of substituted phthalocyanines is being broadly investigated [3,4]. The presence of a substituent on the adjacent aromatic position could lead to a change in the phthalocyanine properties, such as its volume, polarity and charge, and can therefore lead to changes in the aggregation process [5,6].

Several examples involving the synthesis and characterization of substituted phthalocyanines containing chiral groups have been reported [7]. However, there is a lack of studies on chiral secondary alcohols (which can be elegantly obtained by biocatalysis) [8,9] as substituents.

The phthalocyanines modified with chiral secondary alcohols presented here are based on the potential for obtaining different biological properties in structures modified with pure enantiomers. In particular, there may be changes in their interaction with cells, toxicity, aggregation in biological media and other properties, which can result in differences in their photodynamic activity, for example.

In photodynamic therapy, the photosensitizer is released to the target tissue and the region is exposed to light of an appropriate wavelength [10]. Part of the energy is absorbed by the photosensitizer and transferred to oxygen, generating reactive species that promotes cell death [11]. Consequently, the solubility and the interaction with target cells are an important process to be considered for the photosensitizer [12,13].

In this context, we describe the synthesis and characterization of phthalocyanines modified with 1-(4-bromophenyl)ethanol, as both the racemic mixture and with each of its individual (*R*) or (*S*) enantiomers and some biological studies to compare their efficiency as photosensitizers.

## 2. Results and Discussion

### 2.1. Preparation and Structural Characterization

**Scheme 1:**
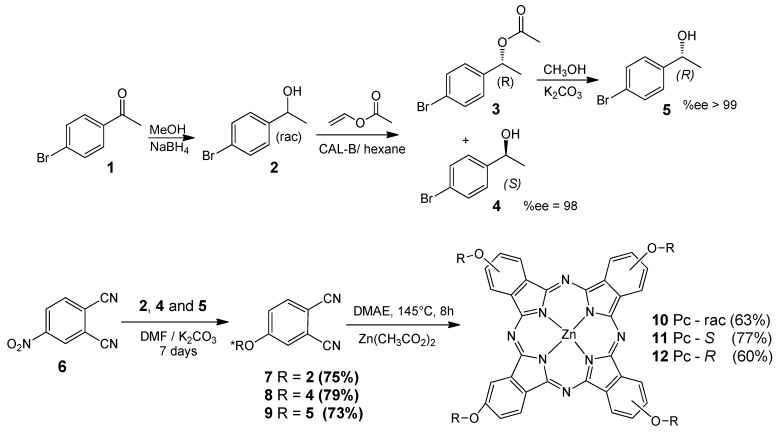
Enzymatic resolution of 1-(4-bromophenyl)ethanol (**1**) and synthesis of phthalocyanines containing chiral substituents.

**Figure 1:**
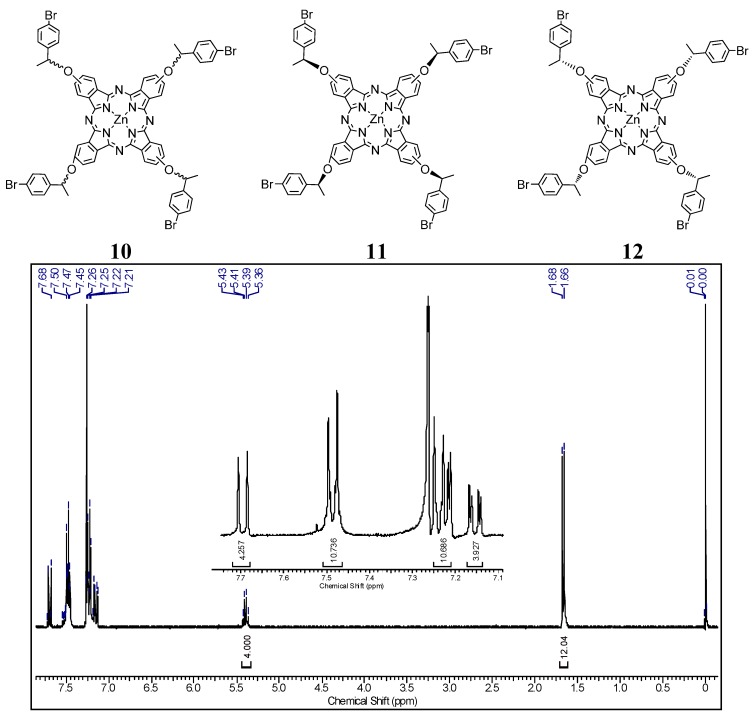
Chemical structure of all synthesized macrocycles and the ^1^H-NMR spectrum of phthlalocyanine **10** in DMSO-*d*_6_.

The first step of the synthesis (Scheme 1) is a well-known chiral resolution of 1-(4-bromophenyl)ethanol [8]. Conditions used were similar to those of a published paper, which helped us to predict the behavior of the isomers (*R*) and (*S*) in the chromatographic purification steps and to determine the absolute configuration of the separated compounds (enantiomeric excess %ee_R_ > 99 and %ee_S_ = 98). All synthesized structures (precursors and phthalocyanines **10**–**12**) were confirmed by FTIR, ^1^H-NMR and mass spectrometry. Figure 1 presents the structure of all synthesized macrocycles and the ^1^H-NMR spectra obtained for phthalocyanine **10**, as an example. The presence (and integration) of signals in the region between 7 to 8 ppm in all ^1^H-NMR spectra confirmed the proportion of aromatic alcohol derivative group attached to the phthalocyanine structure. The methylene and –CH– protons from alcohol peripheral units are well defined in all spectra, with correct integrals, and were observed around 1.5 and 4.0 ppm, respectively. There are no significant differences in the positions and structure of peaks between the spectra of optically active phthalocyanines and the racemic derivative one.

**Figure 2:**
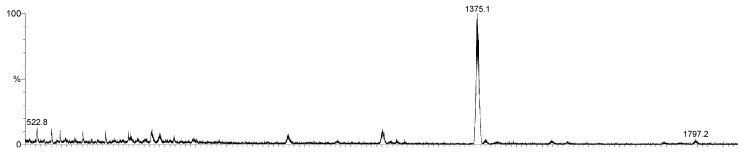
ESI-TOF positive mode mass spectrum of phthlalocyanine **10**.

The FTIR spectra of all compounds presented the absorption bands for C–H and C=C aromatic stretching in the 3.000 and 1.600 cm^−1^ region, the –CH_3_ and –CH stretching between 2.900 to 2.850 cm^−1^ and –C=N relative stretching in 1.340 and 1.220 cm^−1^, confirming the formation of the aromatic phthalocyanine rings. Also, the ESI-TOF mass spectra of compounds presented a molecular ion peak at 1375.1 Da, what is in accordance with the proposed structures, as presented in Figure 2 for compound **10** as example.

**Figure 3:**
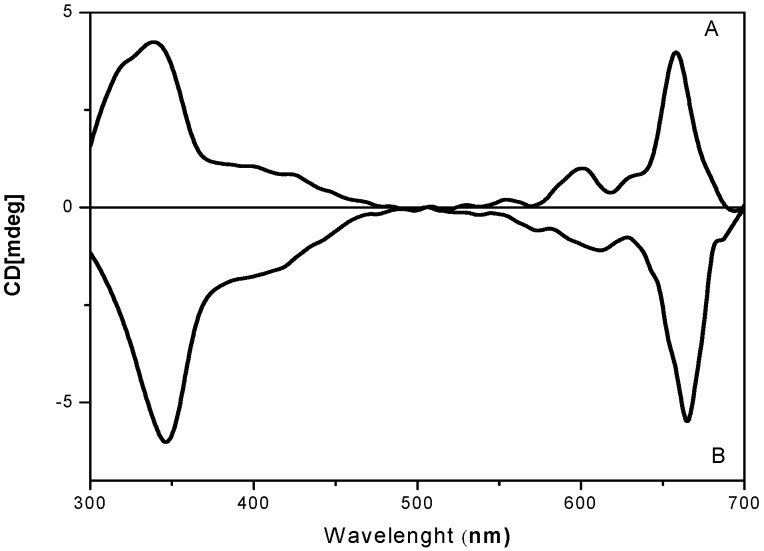
Circular dichroism (CD) spectra of optically active phthalocyanines **11** (**curve B**) and **12** (**curve A**).

In addition, the circular dicroism spectra of phthalocyanines **11** and **12** (Figure 3) confirmed the opposite optical activity properties of the aromatic macrocycle due to the presence of pure enantiomer substituents [14].

### 2.2. UV-Vis Behavior and Aggregation Processes

The absorption spectra of phthalocyanines **10**, **11** and **12** presented absorption bands with maxima around 350 nm and from 600 to 800 nm (Q bands), in DMSO (Table 1), which are typical for these macrocycles in non-aggregated form [15,16]. It was observed that the maximum for the racemic substituent are nearly the same as for the (*R*) and (*S*) configurations, which shows that, in non-aggregated form (low concentrations in DMSO), the configuration of the alcohol moiety does not affect the photophysical properties of the macrocycles.

**Table 1:** Absorption bands, quantum yield of fluorescence (Φ_F_) and of the generation of oxygen reactive species (Φ_Δ_) for phthalocyanine synthesized (all in DMSO).

Pc	λ_max_ (nm)	log Ɛ (cm^−1^·M^−1^, 684 nm)	Log P	Φ_Δ_	Φ_F_
10	356, 615, 684	5.02	1.97 ± 0.01	0.67	0.10
11	356, 615, 684	5.01	2.37 ± 0.09	0.65	0.10
12	356, 615, 684	4.91	2.78 ± 0.02	0.76	0.12

**Figure 4:**
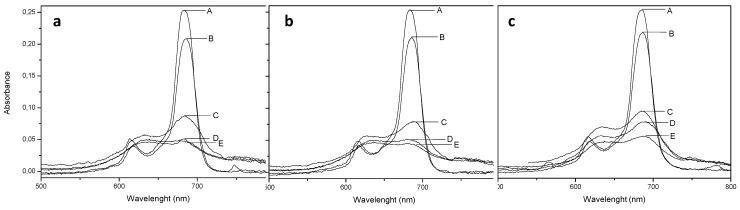
UV-vis absorption spectra of compound **10** (**a**); **11** (**b**) and **12** (**c**), at same concentration, in media with different proportions of DMSO and water (DMSO:water 100:0 (A), 80:20 (B), 60:40 (C), 40:60 (D) and 20:80 (E)).

However, in polar environments, the aggregation processes is intense, promoting a reduction in the relative intensity of the Q bands and a red blue shift in the maximum (Figure 4). Our results have shown that the substituents cannot completely prevent the aggregation process in intense polar media, but this process is different for each phthalocyanine studied. Phthalocyanine **12**, with a (*R*) resolution chiral moiety, presented the absorption band around 680 nm with higher intensity related to the band with maximum at 615 nm in up to 60% of water in DMSO. This higher intensity of the 680 nm band indicates a major presence of monomers than oligomers at this polarity. The compounds **10** and **11**, however, presented a characteristic oligomeric species behavior in mixtures with 40% of water in DMSO, with a maximum shifted to approximately 630 nm.

**Figure 5:**
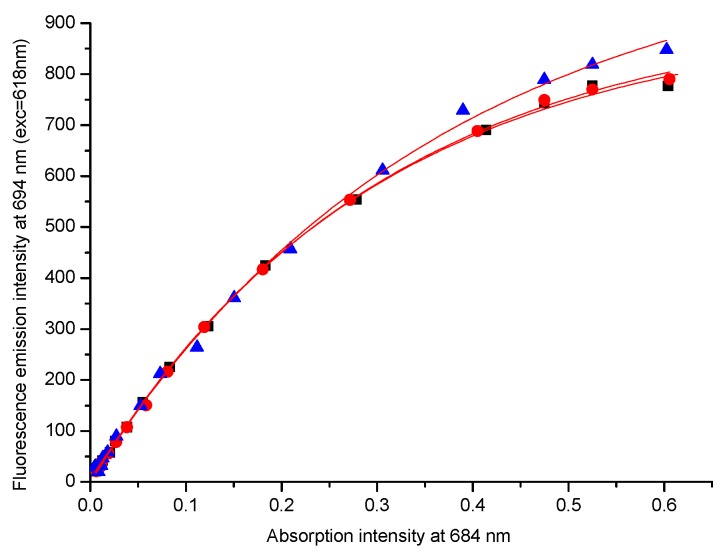
UV-vis absorption intensity at 684 nm and fluorescence emission intensity at 702 nm (λ_exc_ = 618 nm) under the same instrument conditions for compounds **10** (rac, ▲), (**11**, *S*, ●) and (**12**, *R*, **■**), for different concentrations in DMSO, with higher stock solution 5 μmol·L^−1^.

This different behavior shows that the geometry of the substituent can influence the aggregation process of the phthalocyanines, which is generally attributed to the self-facial arrangement of a range of macrocycles [17]. In pure DMSO, the results also suggest differences in the aggregation process. For different concentrations, the intensity of the maximum absorption band (684 nm) were plotted versus the intensity of maximum fluorescence emission band (720 nm, λ_exc_ = 618 nm). For low absorption intensities (low phthalocyanine concentrations, from 0.1 to 2 µmol·L^−1^), a linear relationship between absorption and emission intensities were observed. However, for more concentrated solutions (absorption maximum between 0.4 and 0.7), a nonlinear relationship were verified, suggesting the presence of aggregation (Figure 5).

The phthalocyanine aggregation is due to J aggregates (head-to-tail aggregation) and H aggregates (face-to-face aggregation) [18,19], and the geometry of peripheral substituents can result in different aggregation. Although not very discrepant, this difference can be sufficient to provide differences in their photochemical and photophysical properties, and in their interaction with cells, such as MCF7.

### 2.3. Generation of Oxygen Reactive Species and Fluorescence Quantum Yield

**Figure 6:**
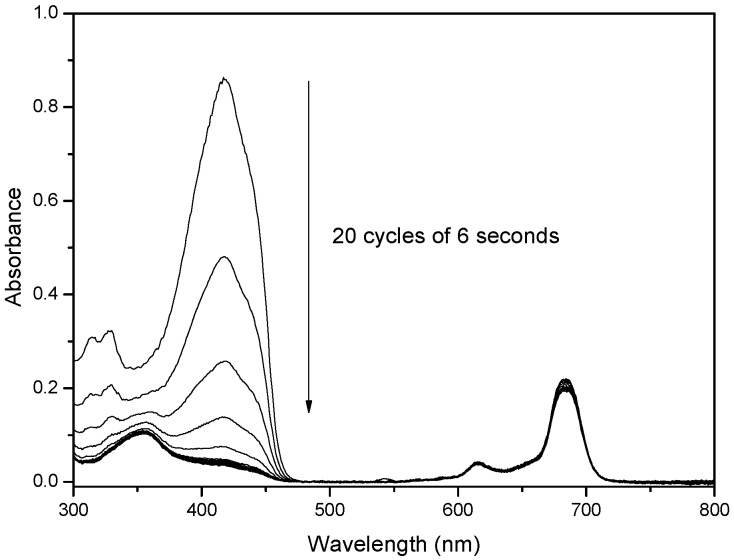
UV-vis spectra of solution containing phthalocyanine (**12**) (absorption ~0.2 in 680 nm) and the DPBF (absorption ~0.9 in 418 nm), under constant irradiation by red LED light, obtained after each 6 s period of irradiation.

Another important aspect in the characterization of photosensitizers for photodynamic therapy is the fluorescence quantum yield (Φ_F_) and its quantum yield (Φ_Δ_) of the generation of oxygen reactive species under visible light irradiation [20,21]. To assess this property, we used an indirect method with diphenylisobenzofuran (DPBF) as chemical quencher, comparing their rate of degradation promoted by each of the synthesized phthalocyanine derivatives and by unsubstituted zinc(II)phthalocyanine (ZnPc) under the same conditions [22,23,24]. The relationship between the rate of degradation of DPBF promoted by each one of these phthalocyanine derivatives, under the same conditions, can be used as an indicator of their photosensitizing properties. For this analysis, a mixture of each phthalocyanine (absorption ~0.2 in 680 nm) and the DPBF (absorption ~0.9 in 418 nm) was irradiated with red LED lamp in 20 cycles of 6 s and the result obtained for compound **12** is showed in Figure 6 as example.

The values obtained for Φ_Δ_ in DMSO and in DMSO-water media differ according to the kind of substituent in each structure, and are in accordance with the demonstrated different tendency of macrocycles to form dimers and oligomers in polar environments. The results have shown that the macrocycle with the (*R*) enantiomer substituent presents a higher quantum generation yield, which is in accordance with its lower tendency to form aggregates under the same conditions compared to the others. This behavior is also observed in more polar environments, like in mixtures of DMSO:water (20:80 to 80:10), where the (*R*) compound also presents a greater rate of decomposition of DPBF compared to others at same conditions.

### 2.4. In Vitro Study

**Figure 7:**
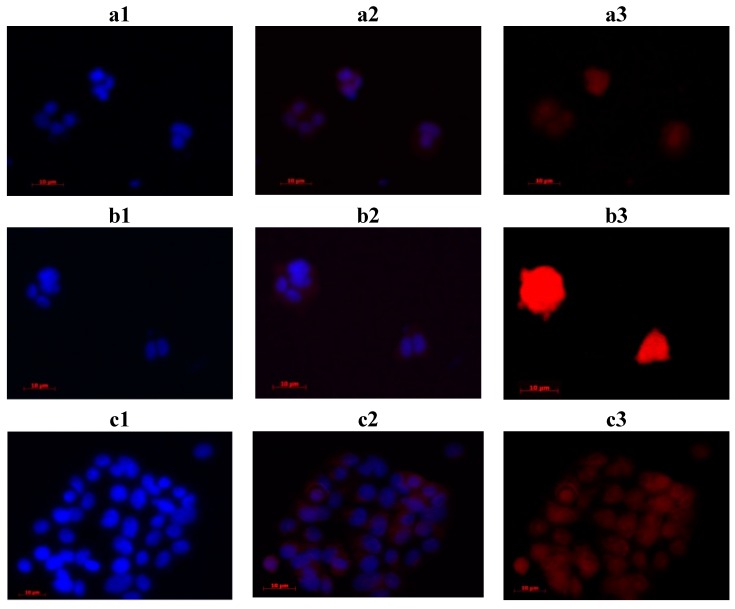
Fluorescence images of the interaction between phthalocyanines (45.0 µM final concentration) **10** (images **a1**–**a3**), **11** (images **b1**–**b3**) and **12** (images **c1**–**c3**) and MCF7 cells (grown on chamber slides at a density of 4 × 10^4^ cells/cm^2^ and treated with 300 nM DAPI solution). The images were produced by 365 nm excitation, using 420 nm (DAPI) and 590 nm (phthalocyanines) emission filter. DAPI was used to following the studied phthalocyanine (red fluorescence) according to the nuclei position. AXIO—observer A1 (Zeiss).

The interaction of phthalocyanines with cells in culture was visualized using fluorescence microscopy. In the fluorescence microscopy experiments, the nuclei of MCF-7 tumor cells were labeled with DAPI (nucleolus) to evaluate the localization of phthalocyanine in the red fluorescence line (Figure 7). This qualitative analysis showed that all three phthalocyanines synthesized here presented an interaction with target cells, with some visual differences between then.

**Figure 8:**
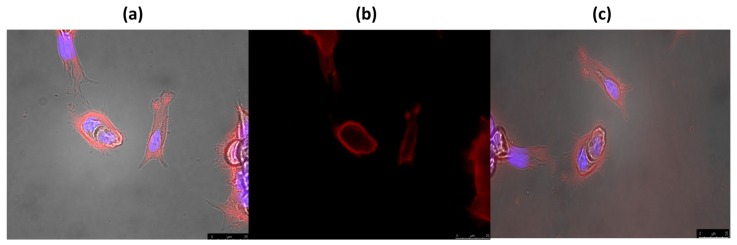
Fluorescence images of the interaction between phthalocyanine **12** (50.0 µM final concentration) and MCF7 cells (**a**,**b**). The images were produced by 365 nm excitation, using 420 nm (DAPI) and 590 nm (phthalocyanine) emission filter. DAPI was used to following the studied phthalocyanine (red fluorescence) according to the nuclei position. (**c**) Presents the same phthalocyanine cell interaction after 10 min of red light irradiation (660 nm, 8 W/cm^2^).

**Figure 9:**
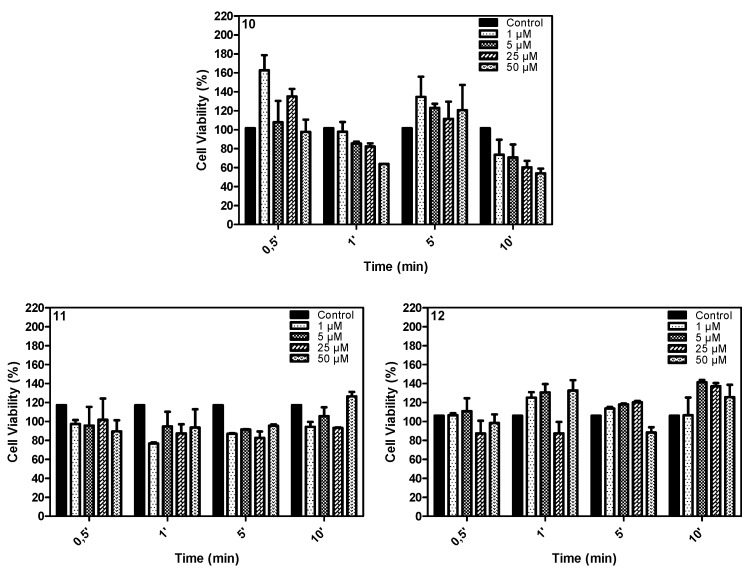
Phototoxic effect of compounds **10**, **11** and **12** on the MCF-7 cell line in different irradiation times (50 μM, 25 μM, 5 μM and 1 μM; 2 h incubation). The control group did not receive the light treatment, only the phthalocyanine.

In detailed fluorescence images (Figure 8a,b), it is possible to visualize the interaction of compound **12** with the MCF7 cell membrane, in a clear red emission circulating the nucleolus labeled with DAPI (blue). Also, even after 10 min of red light PDT treatment (Figure 8c), it is possible to confirm the presence of the macrocyclic compound, with little membrane damage, in agreement with our photodynamic results (Figure 9). The two other phthalocyanines presented the same behavior, accumulating only in MCF7 cell membrane.

**Figure 10:**
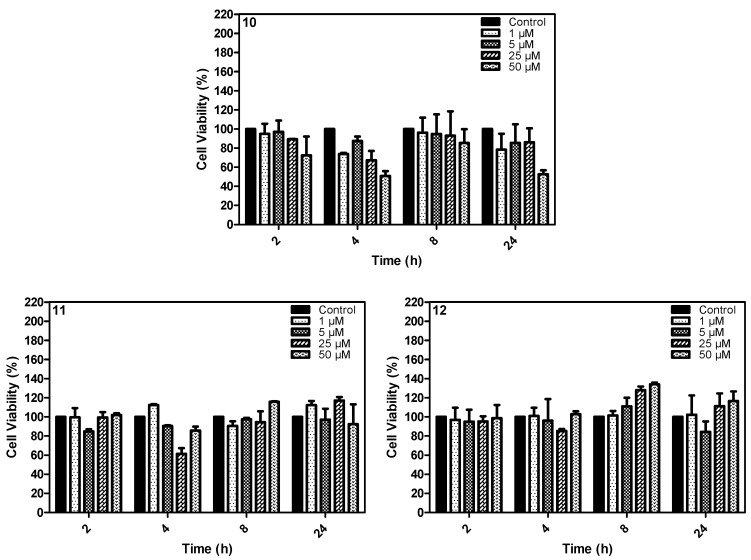
Toxic effect of compounds **10**, **11** and **12** on the MCF-7 cell line after different incubation times and concentrations (50 μM, 25 μM, 5 μM and 1 μM). The control group did not receive the treatment.

The dark toxicity was obtained for all phthalocyanines, evaluating the MCF-7 cell line viability after different incubation periods with or without irradiation. No significant toxicity was observed for all compounds (Figure 10).

The phototoxicity was evaluated for a range of phthalocyanine concentrations (1 μM, 5 μM, 25 μM and 50 μM) under irradiation with a 660 nm LED light (8 W·cm^−2^) for different times (0.5; 1; 5 and 10 min).

The results showed that compounds **11** and **12** presented similar phototoxicity for all concentrations and irradiation times studied. Compound **10**, however, presented a higher phototoxicity, promoting a reduction in about 50% of cell viability after 10 min of irradiation at 50 μM. The observed results can be related with the hydrophilic-hydrophobic difference between compounds, indicated by their partition coefficient values and aggregation behavior in aqueous media.

In our biological protocol, the compounds were dissolved in DMSO and added to RPMI-1640 cell medium to final concentrations ranging from 1 μM to 50 μM. This procedure leads to approximately 2% final DMSO in the aqueous medium, which promotes, as demonstrated in our solubility studies, a high aggregation for all phthalocyanines analyzed, so when we analyze the phototoxicity results, part of better PDT results observed for compound **10** can be explained considering that the racemic mixture has half of the concentration of each enantiomer, what can lead to a reduced aggregation combination in biological medium for this compound when compared to **11** or **12** at similar concentrations. 

However, our results demonstrated that compound **10** presented a higher phototoxicity than observed for both combined pure enantiomers **11** and **12** at the same concentrations, mostly at 25 μM and 50 μM. This result indicates that the presence and distribution of chiral ligands in enantiomeric pure phthalocyanines promote different patterns of cell interaction for each compound, influencing this important step in photodynamic treatment.

Further analyses should be undertaken to understand the influence of chiral ligands on the interactions between phthalocyanines and cells. In addition, in order to minimize the solubility and aggregation problem, these compounds could be encapsulated in bio-carriers to improve their biological properties. In this case, our observation opens a field for investigation to find new photosensitizers for photodynamic therapy.

## 3. Experimental Section

### 3.1. General Information

All reagents and starting materials were purchased from Sigma-Aldrich (St. Louis, MO, USA), except for other specification, and used as received. For ^1^H-NMR spectra (recorded in CDCl_3_ at 500 MHz NMR instrument from Bruker Daltonics Inc. (Billerica, MA, USA) tetramethylsilane was used as internal reference. Flash chromatography was carried out on silica gel (230–400 mesh). Mass spectrometry analyses were recorded on an Electrospray-TOF (ESI-TOF) mass spectrometer from Bruker Daltonics Inc. (Billerica, MA, USA). Conversions and enantiomeric excesses of the enzyme-catalyzed reactions were determined with similar conditions described in [6], using a GC-17A gas chromatograph (Shimadzu Corp, Kyoto, Japan) equipped with a chiral capillary column Chirasil-Dex CB b-cyclodextrin. Circular dicroism spectra of phthalocyanines were recorded on a J-815 instrument (Jasco Inc., Easton, MD, USA). Fluorescence images were produced by 340 nm excitation, using a 580 nm emission filter in a AXIO-observer A1 fluorescence microscope (Carl Zeiss Group, Oberkochen, Germany). DAPI was used its interaction with cell nuclei and its blue fluorescence, which allows following the positions of the studied phthalocyanines (red fluorescence) with respect to the cell nuclei. 

### 3.2. Synthesis

#### 3.2.1. Kinetic Resolution of 1-(4-Bromophenyl)ethanol

To access the enantioenriched alcohols, we employed the enzymatic resolution of secondary alcohols mediated by lipases (Scheme 1). Thus, racemic 1-(4-bromophenyl)ethanol (prepared from 4-bromoacetophenone) was reacted with *Candida antarctica* lipase (CAL-B) and vinyl acetate in hexane. The acetate **3** and the (*S*)-alcohol **4** (95% *ee*) were isolated in 51% and 37% yield, respectively. Ester **3** was then hydrolyzed, leading to the (*R*)-alcohol **5** with 99% *ee*.

Briefly, 1-(4-bromophenyl)-ethanol (**2**, 5.7 mmol), vinyl acetate (11.4 mmol), lipase CAL-B enzyme (8 mg) and hexane (50 mL) were stirred under reflux at 70 °C for 2 h. After cooling, the reaction mixture was concentrated under reduced pressure and the solid purified by column chromatography on silica gel using hexane-ethyl acetate (9:1) and (4:1), yielding 51% of acetate **3** (0.87 mmol, 353.3 mg, relative to the calculated stoichiometric expected mass) and 37% (1.05 mmol, 212.0 mg) of (*S*)-alcohol **4**. The fractions were separated from the (*R*) product and submitted to a hydrolysis reaction whereby 1-(4-bromophenyl)-ethyl acetate (3, 0.82 mmol), potassium carbonate (0.72 mmol) and methanol (50 mL) were added to a flask and stirred at room temperature for 24 h. After the end of the reaction, analyzed by TLC, the methanol was evaporated and the product was extracted with dichloromethane and washed with a saturated solution of ammonium chloride. The organic phase was dried with sodium sulfate, filtered and evaporated. The product was subjected to silica gel chromatography using hexane-ethyl acetate (4:1) to give an 85% yield (0.70 mmol, 140.1 mg) of (*R)*-alcohol **5**.

#### 3.2.2. Reaction of 4-Nitrophthalonitrile with 1-(4-Bromophenyl)ethanol (*R*, *S* and Racemic Forms)

Each optically pure alcohol and the racemate were reacted with commercially available 4-nitrophthalonitrile and an excess of K_2_CO_3_ in DMF, followed by cyclotetramerization to produce phthalocyanine derivatives **10**, **11** and **12** (Scheme 1). Thus, 1-(4-bromophenyl)ethanol (**2**, **4** or **5**, 1.25 mmol), 4-nitrophthalonitrile (6, 1.0 mmol), potassium carbonate (6.4 mmol) and dimethylformamide (DMF, 3 mL) were added to a reaction flask and agitated at room temperature for 7 days. At the end of the reaction, it was analyzed by TLC. The reaction mixture was poured into cold water and extracted three times with dichloromethane. The organic phase was dried with sodium sulfate and the excess solvent was evaporated. The product was subjected to silica gel chromatography using dichloromethane–hexane (2:1) as eluent.

*4-[1-(4-Bromophenyl)ethoxy]phthalonitrile* (**7**) was isolated in 75% yield. FTIR (KBr) u_max_/cm^−1^ 3072 (Ar–H, w), 2994, 2062, 2890 (–CH, –CH_2_, and –CH_3_, w), 2231 (–C≡N, w), 1600, 1498 (-C=C-, s), 1255, 1033 (Ar-O-CH_2_-). ^1^H-NMR (CDCl_3_) 500 MHz: δ 7.62 (d, *J* = 8.73 Hz, 1H, H-13), 7.51 (d, *J* = 8.58 Hz, 2H, H-6 and H-2), 7.22–7.17 (m, 3H, H-3, H-5 and H-10), 7.10 (dd, *J*_1_ = 8.73 Hz, *J*_2_ = 2.64 Hz, 1H, H-14), 5.34 (q, *J*_1_ = 6.39 Hz, 1H, H-7), 1.67 (d, *J*_1_ = 6.39 Hz, 3H, H-8). ESI-TOF *m*/*z* calcd. for C_14_H_14_N_2_O_3_ [M + Na]^+^ 281.0902, found 281.0896.

*4-[S-1-(4-Bromophenyl)ethoxy]phthalonitrile* (**8**) was isolated in 79% yield. FTIR (KBr) u_max_/cm^−1^ 3072 (Ar–H, w), 2994, 2062, 2890 (–CH, –CH_2_, and –CH_3_, w), 2231 (–C≡N, w), 1600, 1498 (-C=C-, s), 1255, 1033 (Ar-O-CH_2_-). ^1^H-NMR (CDCl_3_) 500 MHz: δ 7.62 (d, *J* = 8.73 Hz, 1H, H-13), 7.51 (d, *J* = 8.58 Hz, 2H, H-6 and H-2), 7.22–7.17 (m, 3H, H-3, H-5 and H-10), 7.10 (dd, *J*_1_ = 8.73 Hz, *J*_2_ = 2.64 Hz, 1H, H-14), 5.34 (q, *J*_1_ = 6.39 Hz, 1H, H-7), 1.67 (d, *J*_1_ = 6.39 Hz, 3H, H-8). ESI-TOF *m*/*z* calcd for C_14_H_14_N_2_O_3_ [M + Na]^+^ 281.0902, found 281.0896.

*4-[R-1-(4-Bromophenyl)ethoxy]phthalonitrile* (**9**) was isolated in 73% yield. FTIR (KBr) u_max_/cm^−1^ 3072 (Ar–H, w), 2994, 2062, 2890 (–CH, –CH_2_, and –CH_3_, w), 2231 (–C≡N, w), 1600, 1498 (-C=C-, s), 1255, 1033 (Ar-O-CH_2_-). ^1^H-NMR (CDCl_3_) 500 MHz: δ 7.62 (d, *J* = 8.73 Hz, 1H, H-13), 7.51 (d, *J* = 8.58 Hz, 2H, H-6 and H-2), 7.22–7.17 (m, 3H, H-3, H-5 and H-10), 7.10 (dd, *J*_1_ = 8.73 Hz, *J*_2_ = 2.64 Hz, 1H, H-14), 5.34 (q, *J_1_* = 6.39 Hz, 1H, H-7), 1.67 (d, *J*_1_ = 6.39 Hz, 3H, H-8). ESI-TOF *m*/*z* calcd for C_14_H_14_N_2_O_3_ [M + Na]^+^ 281.0902, found 281.0896.

#### 2.2.3. Syntheses of Optically Active Phthalocyanine **10**, **11** and **12**

The cyclotetramerization was realized in DMAE (dimethylethanolamine) at 145 °C in a closed system. The phthalonitriles **7**, **8** or **9**, zinc acetate and dimethylethylamine (DMAE) were added to the reaction flask, which was subjected to reflux in an inert atmosphere for 8 h. After this, the excess solvent was evaporated and the product was subjected to silica gel chromatography using 2% methanol in dichloromethane as eluent The product purity was confirmed by the presence of a single peak in the corresponding HPLC chromatogram using methanol acetonitrile (1:1) as eluent.

*{tetrakis-[1-(4-Bromophenyl)ethoxy]phthalocyaninato}zinc(II)* (**10**): 63% yield; FTIR (KBr) u_max_/cm^−1^ 3070, 2984, 2067, 2897, 2215, 1620, 1488, 1250, 1093, 742; ^1^H-NMR (DMSO-*d*_6_) 500 MHz: δ 7.13–7.71 (4 m, 28H, Ar), 5.36–5.43 (q, 4H, -CH) and 1.67 (s, 12H, -CH_3_). ESI-TOF *m*/*z* calculated C_64_H_44_Br_4_N_8_O_4_Zn (M) 1374.1; found [M + H] 1375.1;

*{tetrakis-[S-1-(4-Bromophenyl)ethoxy]phthalocyaninato}zinc(II)* (**11**)*:* 77% yield; FTIR (KBr) u_max_/cm^−1^ 3068, 2980, 2065, 2894, 2210, 1622, 1489, 1250, 1090, 740; ^1^H-NMR (DMSO-*d_6_*) 500 MHz: δ 7.13–7.71 (3 m, 28H, Ar), 5.36–5.43 (q, 4H, -CH) and 1.67 (s, 12H, -CH_3_). ESI-TOF *m*/*z* calculated C_64_H_44_Br_4_N_8_O_4_Zn (M) 1374.1; found [M + H] 1373.9;

*{tetrakis-[R-1-(4-Bromophenyl)ethoxy]phthalocyaninato}zinc(II)* (**12**)*:* 60% yield; FTIR (KBr) u_max_/cm^−1^ 3072, 2981, 2060, 2891, 2210, 1624, 1484, 1254, 1090, 739; ^1^H-NMR (DMSO-*d*_6_) 500 MHz: δ 7.13–7.71 (4 m, 28H, Ar), 5.36–5.43 (q, 4H, -CH) and 1.67 (s, 12H, -CH_3_). ESI-TOF *m*/*z* calculated C_64_H_44_Br_4_N_8_O_4_Zn (M) 1374.1; found [M + H] 1375.3.

### 3.3. Photophysical and Photochemical Studies

#### 3.3.1. Singlet Oxygen Quantum Yields

Photogeneration quantum yields of singlet oxygen (Φ_Δ_) were obtained by an indirect method, using diphenylisobenzofuran (DBPF) [14,15] as chemical quencher. Typically, a mixture of the phthalocyanine (absorption ~0.2 at 680 nm in DMSO) and the DPBF (absorption ~0.9 at 418 nm in DMSO) was irradiated with red LED lamp in 20 cycles of 6 s. The Φ_Δ_) values were determined using zinc phthalocyanine (ZnPc) as standard (Equation (1)):

(1) $$\Phi_{∆}=\Phi_{∆}^{ZnPc}\frac{w.I_{abs}^{ZnPc}}{w^{ZnPc}.I_{abs}}$$

where (**Φ_Δ_*^ZnPc^***) is the singlet oxygen quantum yield for the ZnPc (Φ_Δ_ = 0.67 in DMSO [10]); **w** and **w^ZnPc^** is the DPBF photobleaching rates in the presence of the phthalocyanine and ZnPc, respectively; **I_abs_** and **I_abs_^ZnPc^** are the light absorption values for phthalocyanines (λ = 660 nm) and ZnPc (λ = 660 nm), respectively.

#### 3.3.2. Fluorescence Quantum Yields

Fluorescence quantum yields (Φ*_F_*) of all compounds were determined by comparative method using the following the equation (Equation (2)):

(2) $$\Phi_{F}=\Phi_{F}^{ZnPc}\frac{F.A_{ZnPc}.\eta^{2}}{F_{ZnPc}.A.\eta_{ZnPc}^{2}}$$

were **F** and **F_ZnPc_** are the areas under the fluorescence emission curves of the phthalocyanines and the standard, respectively; **A** and **A_ZnPc_** are the relative absorbance of the sample and standard at the excitation wavelength, respectively; **η^2^** and **η^2^_ZnPc_** are the refractive indices of the solvents for the sample and standard, respectively. The unsubstituted zinc phthalocyanine (ZnPc) was employed as standard. The solutions were prepared in DMSO with absorbance ranged between 0.05 and 0.1 at the excitation wavelength (λ_exc_ = 618 nm).

#### 3.3.3. Determination of Partition Coefficients (P_O/W_)

Five milliliters of 10 µM solution from each phthalocyanine derivative was prepared in *n*-octanol. The UV-Vis spectrum was sampled. Then, 5 mL of water was added to the solution and the container was stirred for 30 min. Centrifugation (5 min at 5000 rpm) enabled a phase separation and the organic phase was sampled again. The partition coefficient was obtained from the difference in the phthalocyanine absorption intensity (in 650 nm region) in both stages. At least three independent measures were performed and the corresponding P_O/W_ value was taken as the overall average.

### 3.4. Cell Interaction Study 

#### 3.4.1. Cell Culture

The human breast carcinoma line (MCF-7) cells were cultured in 75 cm^2^ bottles with RPMI-1640 medium supplemented with 10% fetal bovine serum, 2 mL insulin purified from bovine pancreas, sodium pyruvate (0.5 mM) and non essentials amino acids: l-alanine (0.89 g/L), l-asparagine H_2_O (1.50 g/L), l-aspartic acid (1.33 g/L), l-glutamic acid (1.47 g/L), glycine (0.75 g/L), l-proline (1.15 g/L) and l-serine (1.05 g/L). The samples were incubated at 37 °C in an atmosphere of 5% CO_2_ and 80% on reaching confluence were trypsinized and reinoculated onto plates at a density of 4 × 10^4^ cells/cm^2^ (fluorescence microscopy) and 2.48 × 10^4^ (cell viability test) were plated for experiments.

#### 3.4.2. Fluorescence Microscopy

Human breast epithelial cells MCF-7 (adenocarcinoma, estrogen receptor +; ATCC) were incubated in RPMI-1640 medium supplemented with 10% fetal bovine serum (Invitrogen Corp, Carlsbad, CA, USA), 10.0 μg/mL insulin purified from bovine pancreas, 100 U/mL penicillin and 10.0 µg/mL streptomycin. MCF-7 cells were grown on chamber slides at a density of 4 × 10^4^ cells/cm^2^ and then treated with 300 nM DAPI solution (4ʹ,6-diamidino-2-phenylindole, Invitrogen Corp, Carlsbad, CA, USA) at 37 °C for 30 min. The cells were then washed with phosphate buffered saline (PBS: 137 mM NaCl and 2.7 mM KCl in 10 mM phosphate buffer at pH 7.4) and incubated with 45 µM phthalocyanines (DMSO) in dark. After 1-h treatment, the cells were pre-fixed with 30% and 50% ethanol diluted in cells medium (*v*/*v*) followed by a 100% ethanol post-fixation. The cells were then washed with PBS. The fluorescence images were produced by 365 nm excitation, using 420 nm (DAPI) and 590 nm (phthalocyanines) emision filter in an AXIO—observer A1 (Zeiss) using a 40× objective and also 590–650 nm excitation and 662–733 nm emission filter in an AF6000 Leica fluorescence microscope using a 63× oil immersion objective.

#### 3.4.3. Cell Viability—MTT

Cell viability assays are important to show the toxicity of a compound. We conducted a MTT colorimetric test to assess cell viability. For this, cells were plated in 96-well plates with 2.48 × 10^4^ density and incubated at 37 °C in an atmosphere of 5% CO_2_. The phthalocyanines were added at 1, 5, 10, 25 and 50 μM, and the cell viability was analyzed in 2, 6, 24 and 48 h. For photodynamic tests, the cells were incubated with phthalocyanines at 50 μM, for 2 h, and the plates were divided into control (no irradiation) and treated (irradiated). The red LED light irradiation (660 nm, 8 W/cm^2^) was applied in period of 0.5, 1.5 and 10 min. The cells viability was analyzed after 2, 6 and 24 h. After the time set for the treatment, was added 30 μL of MTT solution, and the cells were again incubated for one hour. Thereafter the whole solution was removed from the wells and 150 μL of DMSO was added to solubilize formazan crystals formed from the reduction of MTT salt. Viability was measured by ELISA reader which measures the absorbance of each well of the plate. With the values obtained we performed the following calculation: % viable cells = (absorbance control/treated absorbance) × 100.

## 4. Conclusions

A phthalocyanine derivative with an (*R*)-1-(4-bromophenyl)ethanol substituent presented a lower tendency to aggregation than similar phthalocyanine derivatives under the same conditions, resulting in a higher yield in the generation of reactive oxygen species. The results obtained by fluorescence microscopy and cell viabiliity have shown that these phthalocyanines interact in different ways with mammary MCF-7 cells. Therefore, our results indicate that the photochemical and biological properties of phthalocyanines with chiral ligands should be evaluated separately for each isomeric species.
